# Skeletal convergence in thunniform sharks, ichthyosaurs, whales, and tunas, and its possible ecological links through the marine ecosystem evolution

**DOI:** 10.1038/s41598-023-41812-z

**Published:** 2023-10-04

**Authors:** Ryosuke Motani, Kenshu Shimada

**Affiliations:** 1 Department of Earth and Planetary Sciences, University of California, Davis, One Shields Avenue, Davis, CA 95616 USA; 2 Department of Environmental Science and Studies, DePaul University, 1110 West Belden Avenue, Chicago, IL 60614 USA; 3 Department of Biological Sciences, DePaul University, 2325 North Clifton Avenue, Chicago, IL 60614 USA; 4 Sternberg Museum of Natural History, Fort Hays State University, Hays, KS 67601 USA

**Keywords:** Ecology, Ecology, Evolutionary ecology, Palaeoecology, Evolution, Palaeontology

## Abstract

Tunas, lamnid sharks, modern whales, and derived ichthyosaurs converged on the thunniform body plan, with a fusiform body, lunate caudal fin, compressed peduncle, and peduncle joint. This evolutionary convergence has been studied for a long time but little is known about whether all four clades share any skeletal characteristics. Comparisons of vertebral centrum dimensions along the body reveal that the four clades indeed share three skeletal characteristics (e.g., thick vertebral column for its length), while an additional feature is shared by cetaceans, lamnid sharks, and ichthyosaurs and two more by lamnid sharks and ichthyosaurs alone. These vertebral features are all related to the mechanics of thunniform swimming through contributions to posterior concentration of tail-stem oscillation, tail stem stabilization, peduncle joint flexibility, and caudal fin angle fixation. Quantitative identifications of these features in fossil vertebrates would allow an inference of whether they were a thunniform swimmer. Based on measurements in the literature, mosasaurs lacked these features and were probably not thunniform swimmers, whereas a Cretaceous lamniform shark had a mosaic of thunniform and non-thunniform features. The evolution of thunniform swimming appears to be linked with the evolution of prey types and, in part, niche availability through geologic time.

## Introduction

**Figure 1:**
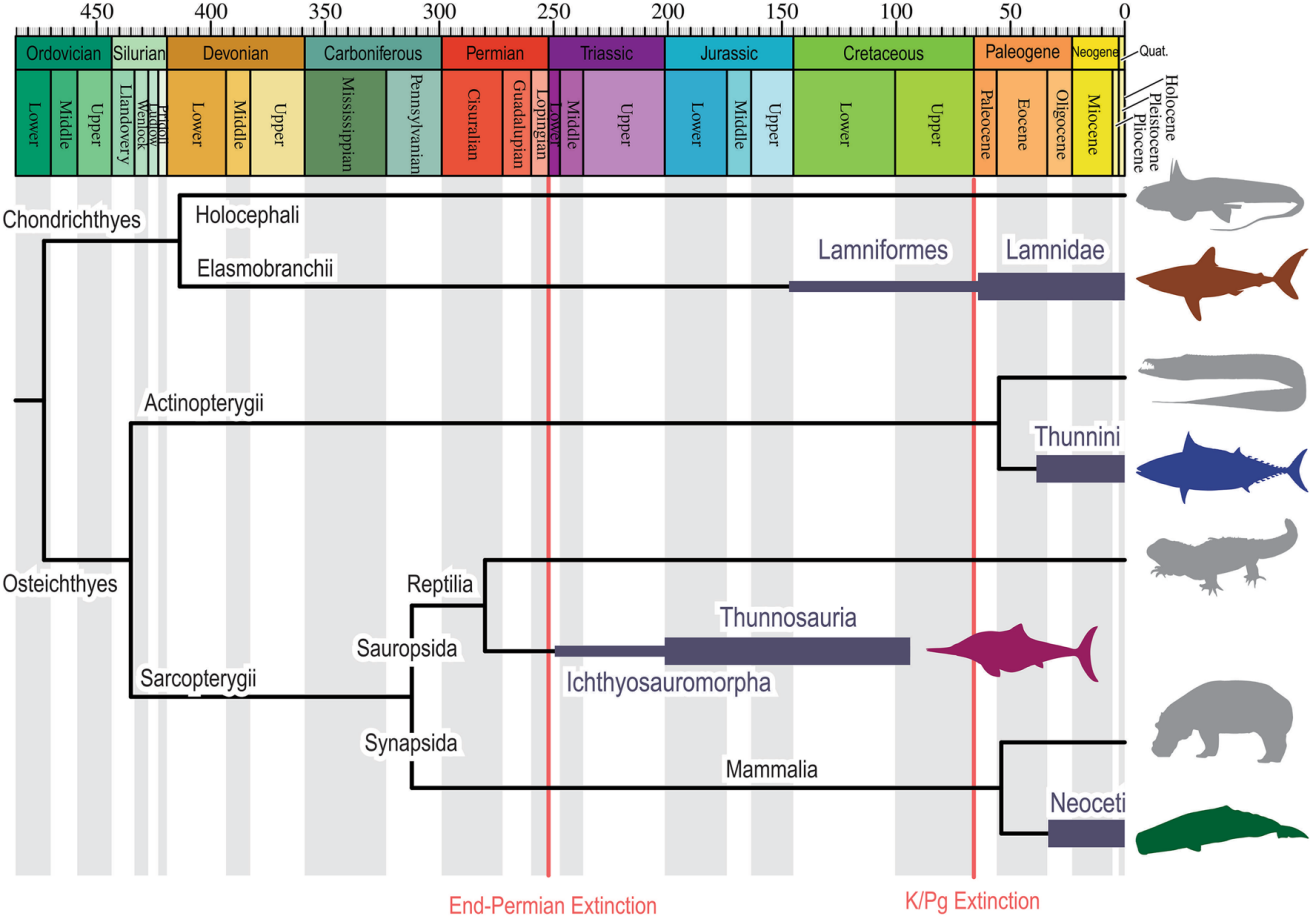
Abbreviated phylogenetic distribution of thunniform vertebrates with their approximate stratigraphic distributions. Node ages are based on recommendations by timetree.org. Ranges of thunniform clades are based on the fossil record (e.g., paleobioldb.org).

Thunniform vertebrates represent an iconic example of evolutionary convergence in which body plan and swimming style of large cruising marine vertebrates exhibit tuna-like features^1^. Their phylogenetic spectrum is arguably the broadest among evolutionary convergences known in vertebrates (Fig. 1), encompassing tunas (Thunnini, Actinopterygii), lamnid sharks (Lamnidae, Chondrichthyes), modern whales (Neoceti, Mammalia), and derived ichthyosaurs (Thunnosauria, Reptilia)^2–4^. No thunniform vertebrates are known before the end-Permian mass extinction, making them one of the modern ecological components that were added after the extinction event (Fig. 1).

The thunniform body plan is characterized by several features, including a fusiform body, compressed peduncle with a pair of keels, lunate caudal fin, and an extra joint between the peduncle and the caudal fin, often called the peduncle joint^5–8^. These characteristics are all related to cruising efficiency, such as the body and tail shapes reducing the drag and maximizing thrust^5,6^, the keeled peduncle reducing negative effects of streamwise vortices^7^, and the peduncular joint adjusting the angle of attack of the caudal fin, concomitantly improving propulsive efficiency^8^.

**Figure 2:**
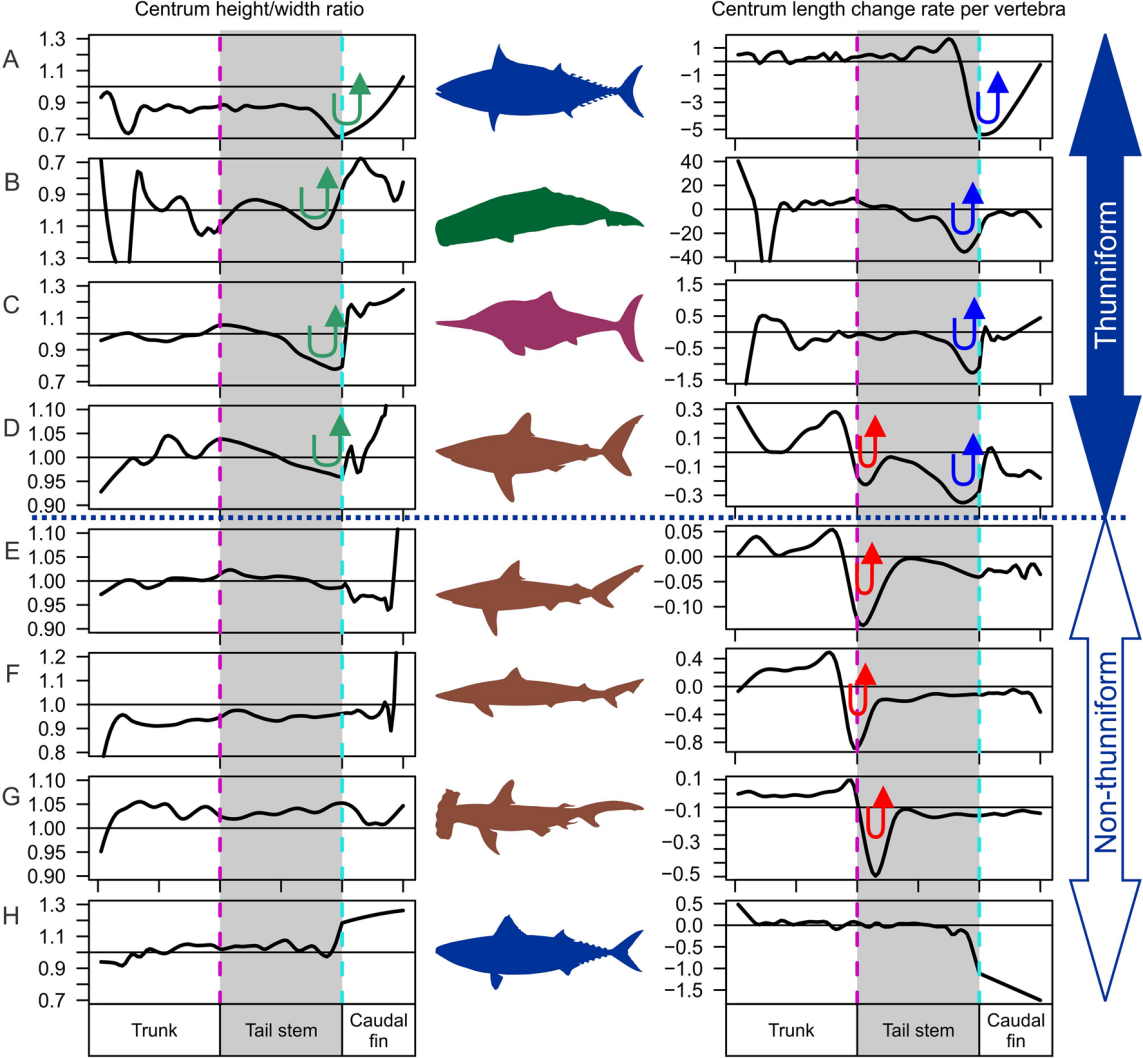
Centrum size and shape along the body in selected thunniform and non-thunniform marine vertebrates. (**A**) *Euthynnus alletteratus* (scombrid bony fish). (**B**) *Physeter macrocephalus* (sperm whale). (**C**) *Ophthalmosaurus icenicus* (ichthyosaur). (**D**) *Lamna nasus* (lamnid shark). (**E**) *Carcharhinus leucas* (carcharhinid shark). (**F**) *Galeorhinus galeus* (triakid shark). (**G**) *Sphyrna tudes* (sphyrnid shark). (**H**) *Scomber japonicas* (scombrid bony fish). Blue arrow: centrum shortening rate increase and decrease at the peduncle joint. Green arrow, centrum widening and narrowing at the peduncle joint. Red arrow, centrum shortening due to diplospondyly in sharks. Top four are thunniform swimmers whereas the rest are not. For teleosts, the hypural is excluded.

The thunniform convergence is not limited to what is observed externally. For example, the centrum shape of caudal vertebrae changes in a common pattern near the peduncle joint in cetaceans and ichthyosaurs^9^. The vertebral centra in the posterior tail stem are compressed parallel to the plane of tailbeat, i.e., dorsoventrally compressed in ichthyosaurs and laterally in cetaceans, but within the caudal fin, they are compressed perpendicular to that direction, parallel to the plane of the caudal fin^9,10^ (Fig. 2B, C, green arrows). This was interpreted to stabilize the tail stem during oscillation while keeping the caudal fin flat^9,10^. However, this feature has not been examined in the other two clades. The tail stem in this context is the part of the tail anterior to the caudal fin.

**Figure 3:**
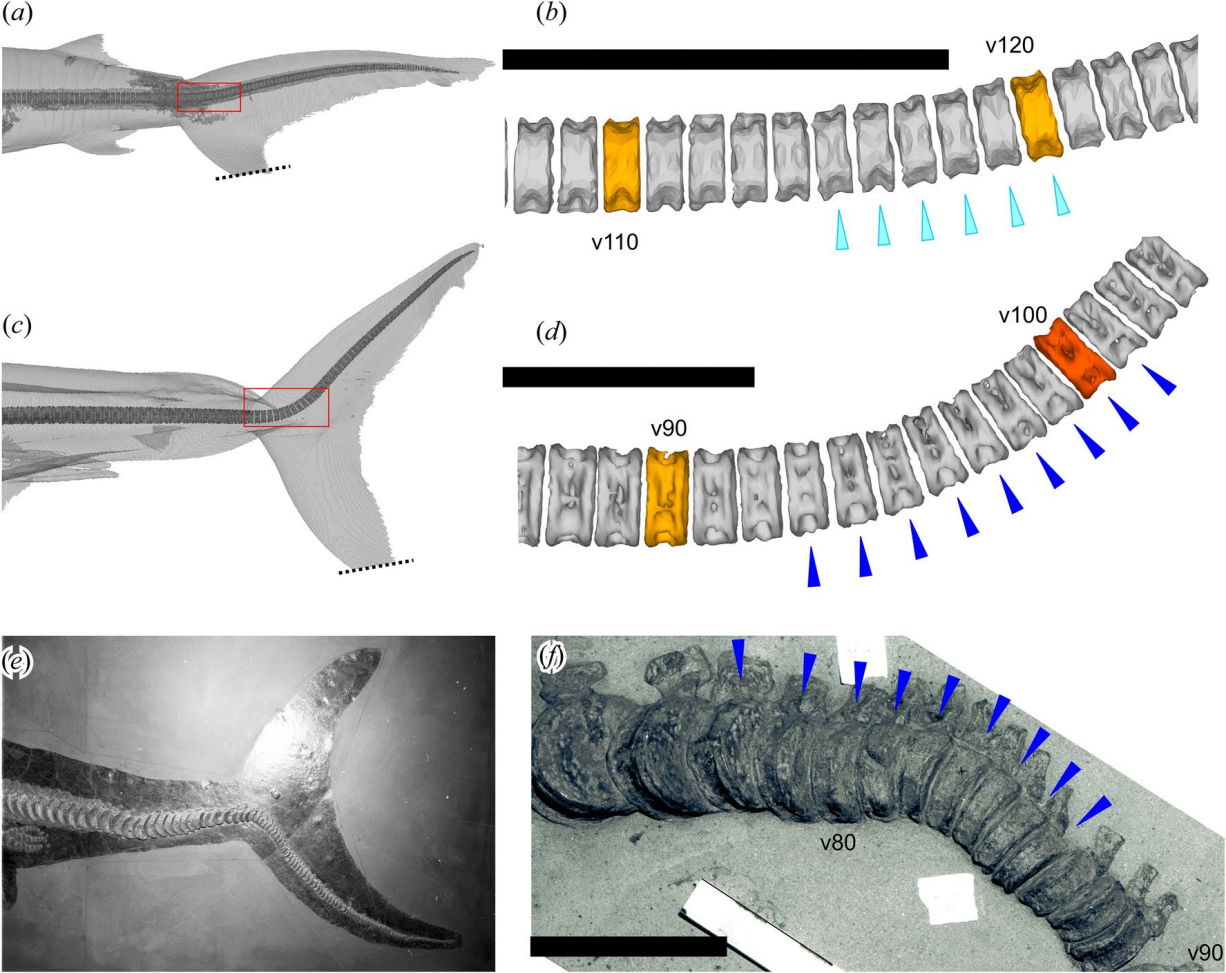
Posterior vertebral column in selected shark and ichthyosaur taxa. (**a**) heterocercal caudal fin of *Carcharhinus leucas* (carcharhinid shark: Rijksmuseum van Natuurlijke Historie RMNH.PISC.24271). (**b**) calcified centra at the tailbend region for the same. (**c**) heterocercal caudal fin of *Lamna nasus* (lamnid shark: Zoölogisch Museum Amsterdam ZMA.PISC.116165). (**d**) calcified centra at the tailbend region for the same. (**e**) hypocercal caudal fin of *Stenopterygius quadriscissus* (ichthyosaur: SMNS 16811). (**f**) ossified centra at the tailbend region for the same species (SMNS 14846). Dark blue triangles, conspicuously wedge-shaped centra. Light-blue triangles, weakly wedge-shaped centra. Scale bars are 5 cm.

Of the four thunniform clades, lamnid sharks and ichthyosaurs share a common caudal fin design where the vertebral column extends into one of the caudal fin lobes, although it is in the dorsal lobe in sharks (heterocercal condition) and the ventral lobe in ichthyosaurs (hypocercal condition)^11,12^ (Fig. 3c–f). These conditions require deflection of the vertebral column at the onset of the caudal fin, referred to as the tailbend, formed by a series of conspicuously wedge-shaped vertebral centra in both ichthyosaurs and lamnid sharks^11,12^. However, it is not fully known if the tailbend structure is present in non-thunniform sharks, where such knowledge would allow the identification of thunniform swimmers among fossil sharks based on wedge-shaped centra.

Previous studies on thunniform convergence benefited greatly from pairwise comparisons^4,9,11^, but it is unknown if all four clades share any common internal features. Computerized tomographic (CT) images of many shark skeletons are now openly available^13^, allowing quantitative comparisons of vertebral columns with tunas, cetaceans, and ichthyosaurs. The purpose of the present paper is to compare the vertebral centra of all four clades to examine if they all share some common features that are lacking in outgroups comprising non-lamnid sharks, non-Thunnini scombrids, and non-thunnosaurian ichthyosaurs. Cetaceans are not included in outgroups given that the timing of transition to thunniform swimming is ambiguous. Four hypotheses are tested: (1) all four clades have thick vertebral columns for their lengths; (2) the abrupt change of centrum shape at the onset of the caudal fin is shared by all four clades; (3) centrum size changes along the caudal vertebral column in the same way in all four clades; and (4) the tailbend formed by a series of conspicuously wedge-shaped vertebral centra is unique to lamnids among sharks and thus constitute a valid thunniform feature.

## Results

### Relative width of vertebral centra in tail stem

**Figure 4:**
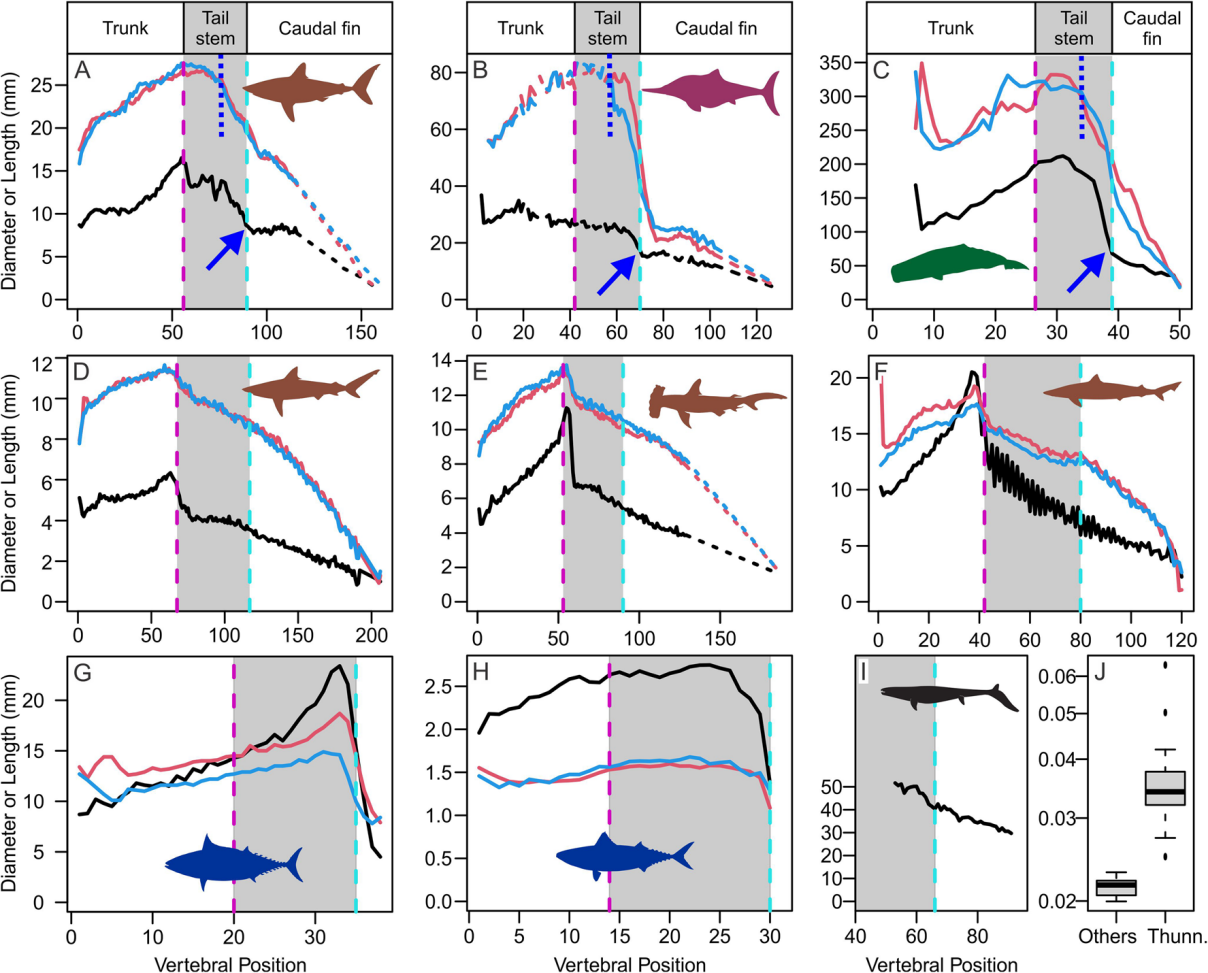
Centrum dimensions along the vertebral columns in selected swimming vertebrates. (**A**) *Lamna nasus* (lamnid shark). (**B**) *Ophthalmosaurus icenicus* (ichthyosaur). (**C**) *Physeter macrocephalus* (sperm whale)^41^. (**D**) *Carcharhinus leucas* (carcharhinid shark). (**E**) *Sphyrna tudes* (sphyrnid shark). (**F**) *Galeorhinus galeus* (triakid shark). (**G**) *Euthynnus alletteratus* (scombrid bony fish). (**H**) *Scomber japonicas* (scombrid bony fish). (**I**) *Platecarpus* sp. (mosasaur)^22^. (**J**) boxplot of relative centrum diameter to the body for thunniform (Thunn.) and other vertebrate swimmers (Others). Curves in (**A**)–(**J**) are width (red), height (light blue), and length (black). Centrum shortening suddenly slows down at blue arrows in (**A**)–(**C**).

The vertebral column is thick for its length in thunniform swimmers compared with other fully aquatic vertebrates examined (Fig. 4J), except for thresher sharks and some dorsoventrally flattened species, such as angel sharks and wobbegongs. The width of the widest centrum is on average 9.1% (6.0–13.5) of the tail-stem length (= the cumulative sum of centrum lengths from the pelvic girdle to the onset of the caudal fin) in thunniform swimmers but 4.9% (3.7–5.6) in the others. Likewise, the maximum centrum width is on average 3.7% (2.5–6.3) of the cumulative centrum lengths from the neck to the onset of the caudal fin in thunniform swimmer whereas it is 2.1% (2.0–2.3) in the others. The difference of mean ratios between thunniform swimmers (n = 19) and non-thunniform outgroup (n = 19) is significant in both cases based on ANOVA of log-transformed ratios (*p* < 0.001, F = 33.21 for the former, and *p* < 0.001, F = 23.4 for the latter).

### Change of centrum shape at the onset of the caudal fin

The abrupt change of the centrum compression axis at the onset of the caudal fin, explained earlier for ichthyosaurs and cetaceans, is also present in the porbeagle shark (*Lamna nasus*: Fig. 2D, green arrow) and little tunny (*Euthynnus alletteratus*: Fig. 2A, green arrow). This feature is absent in other sharks (Fig. 2E–G), except possibly in the bull shark (*Carcharhinus leucas*: Fig. 2E) and chub mackerel (*Scomber japonicas*: Fig. 2G), in which their centra do not widen greatly anterior to the caudal fin unlike in thunniform species.

### Change of centrum size along the caudal vertebral column

All four clades share a common pattern of how the centrum length changes near the onset of the caudal fin, where the rate of centrum shortening accelerates anterior to the caudal fin but suddenly decelerates at the onset of the fin (blue arrows in Fig. 2A–D and 4A–C). *Scomber japonicus* would exhibit this pattern if the hypural plate, which is a large plate of bone from the fusion of caudal fin vertebrae typical of teleosts, is included, whereas the same pattern exists in *Euthynnus alletteratus* without the hypural. In lamnids and ichthyosaurs, centra not only seize to shorten before the onset of the caudal fin but also starts lengthening slightly after the onset (Figs. 2C, D, 4A, B). Sharks other than lamnids do not exhibit such abrupt size shifts, but rather the centrum length continuously decreases gradually through the tail (Figs. 2E–G, 4D–F). Mosasaurs also seem to exhibit the same pattern as in non-lamnid sharks according to published data (Fig. 4I)^14^.

Cetaceans, lamnids, and thunnosaurian ichthyosaurs share another common feature—i.e., the centrum diameter, both laterally and dorsoventrally, decreases slowly in the anterior tail stem but rapidly in the posterior tail stem (Fig. 4A–C, vertical blue dotted lines form the boundaries), thus shortening the effective tail stem where the decrease is obvious. This feature is prevalent among teleost fishes but not in other outgroup taxa. In other sharks, the centrum size continuously decreases gradually in the tail stem except for the abrupt shift at the onset of diplospondyly (having two centra per body segment) (Fig. 4D–F).

### Tailbend and conspicuously wedge-shaped centra

**Figure 5:**
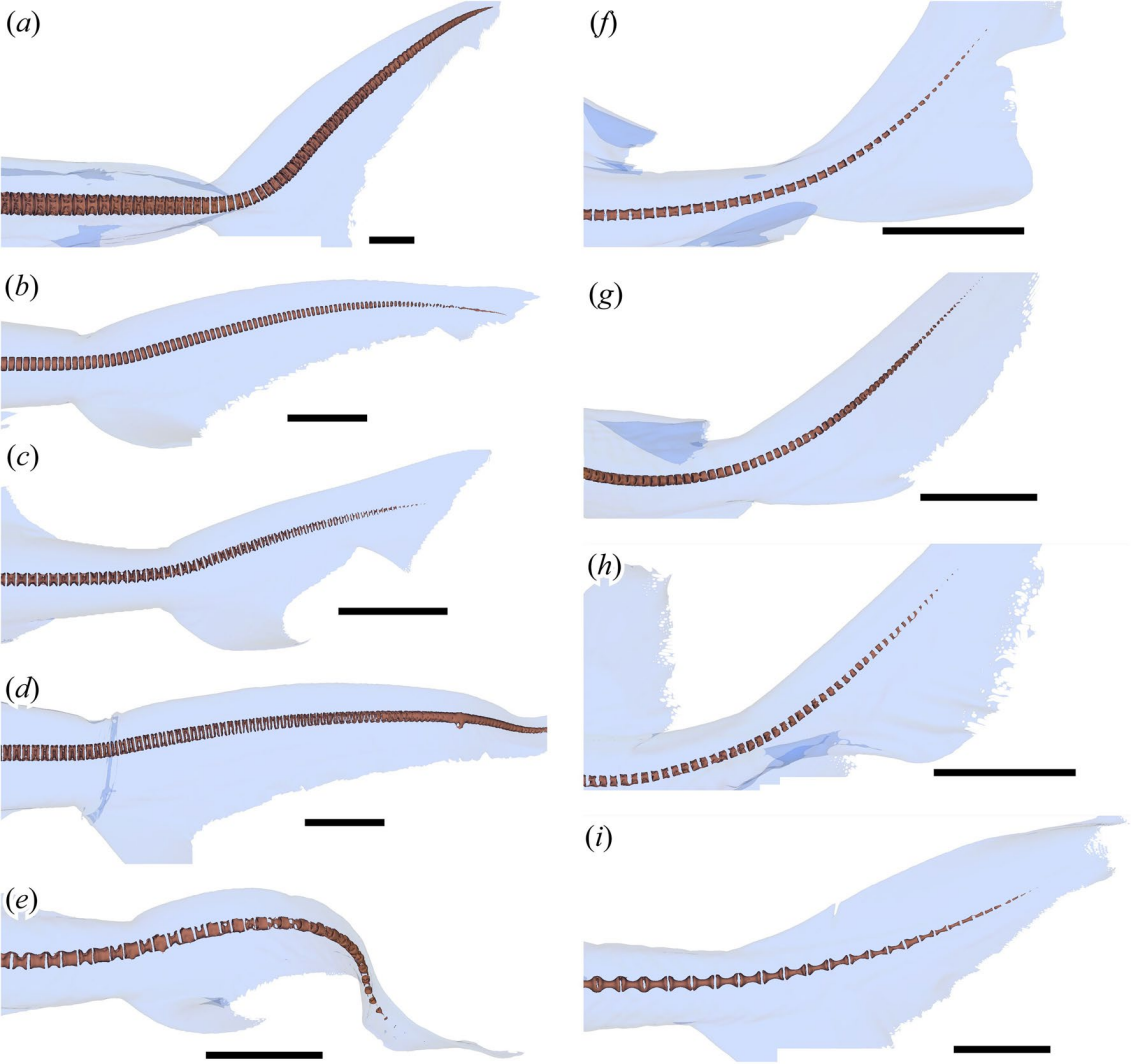
Calcified vertebral centra and outlines of the caudal fin in selected sharks as captured in CT images. (**a**) *Lamna nasus* (ZMA.PISC.116165). (**b**) *Carcharhinus leucas* (RMNH.PISC.24271). (**c**) *Galeorhinus galeus* (RMNH.PISC.36345). (**d**) *Sphyrna tudes* (ZMA.PISC.109128). (**e**) *Eusphyra blochii* (ZMA.PISC.108685). (**f**) *Heterodontus francisci* (ZMA.PISC.109128). (**g**) *Ginglymostoma cirratum* (ZMA.PISC.108710). (**h**) *Eucrossorhinus dasypogon* (RMNH.PISC.7411). (**I**) *Dalatias licha* (ZMA.PISC.112272). Scales bars are 5 cm.

The tailbend is formed in three different ways in the 63 shark species examined. The first type is known only in lamnids, which have a successive series of conspicuously wedge-shaped centra constituting about 45° of tailbend angle in total, and about 5° per centrum on average (Fig. 3c, d, 5a), resembling those of thunnosaurian ichthyosaurs. The intervertebral cartilage does not contribute to the tailbend angle. The second type completely lacks wedge-shaped centra; yet, there is up to about 45° of tail deflection formed by uniform curvature of the vertebral column (Fig. 5f–i). To be precise, wedge-shaped calcified centra may be irregularly present but the wedge angles do not contribute to the tail curvature, i.e., the dorsal curvature of the tail vertebral column is uniform whether there is a wedge or not because any wedge angle from a centrum is canceled out by adjacent intervertebral cartilage which are wedged in the opposite direction (e.g., Fig. 5g, h). This type is exemplified by the kitefin shark (*Dalatias licha*), horn shark (*Heterodontus francisci*), tasselled wobbegong (*Eucrossorhinus dasypogon*), nurse shark (*Ginglymostoma cirratum*), and common thresher (*Alopias vulpinus*). The third type is in-between the first two, where there are a few successive weakly wedge-shaped centra or a single conspicuously wedge-shaped centrum, which form a small tailbend of up to about 15° together with intervertebral cartilages (Fig. 5c–e). These weakly wedge-shaped centra each provide a few degrees of bending angle. This type is exemplified by the school shark (*Galeorhinus galeus*), winghead shark (*Eusphyra blochii*), and smalleye hammerhead (*Sphyrna tudes*)*. Carcharhinus leucas* is considered here an extreme variant of this third type, in which there are six weakly wedge-shaped centra, contributing to about 15° of tailbend angle (Figs. 3a, b, 5b).

## Discussion

**Figure 6:**
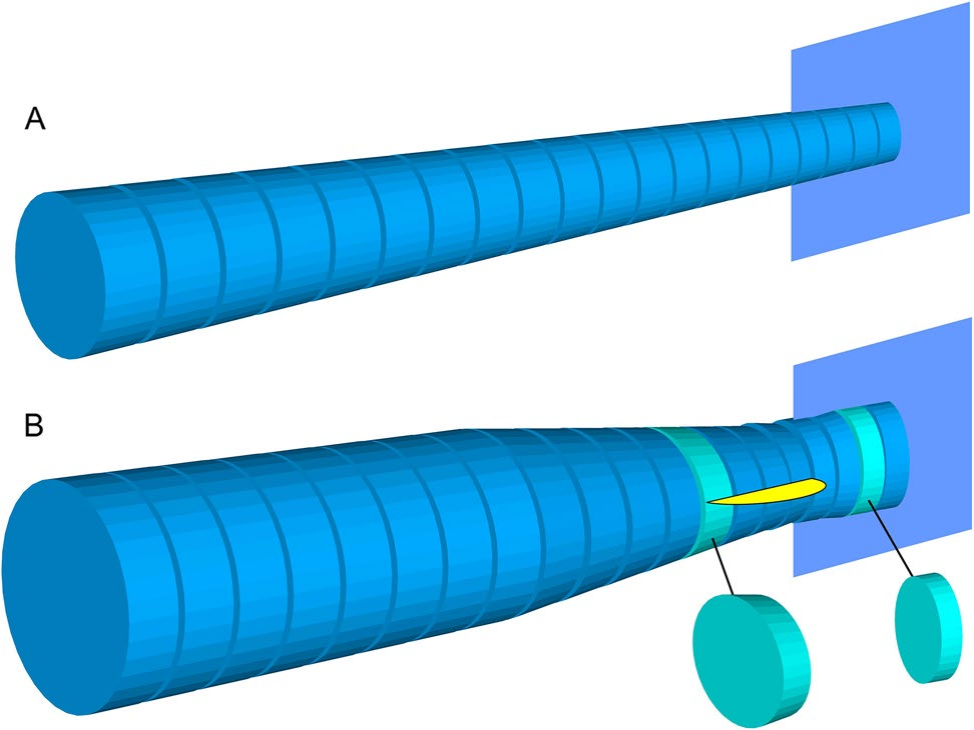
Exaggerated schematic drawings of caudal centra, illuminating the first four features in Table 1. (**A**) Non-thunniform swimmers. (**B**) Thunniform swimmers. Each blue vertical plane indicates the approximate position of the caudal fin, and the peduncular region is situated immediately anterior to the caudal fin. The yellow flap in (**B**) shows the approximate positioning of the horizontal peduncular keel. Compared to non-thunniform swimmers (**A**), thunniform swimmers (**B**) have: (1) an overall thicker vertebral column for length; (2) a more abrupt change of centra compression axis around the peduncle as illuminated by light blue centra in (**B**); (3) shorter centra near or immediately after the peduncle; and (4) thicker anterior caudal column without a drastic decrease in the size of centra until about halfway in the tail. The drawings are for forms with horizontal tailbeats.

The present study reveals that three vertebral features are shared by the four clades of thunniform vertebrates (Fig. 6), with additional three shared by subsets of the four (Table 1). These features reflect the mechanics of thunniform swimming. Simple beam theory suggests that the diameter of the vertebral centra is correlated with regional bending strengths of the vertebral column, so the thick vertebral column provides stability of the region. Additionally, by keeping the vertebral centra large in the anterior caudal region and then rapidly reducing their size posteriorly toward the caudal fin (Fig. 4, vertical blue dotted lines; Fig. 6), the most flexible region of the tail-stem column is concentrated posteriorly in the tail. This skeletal pattern agrees with the observation that the tail oscillation is restricted more posteriorly in thunniform swimmers^1,15^. The abrupt change of the centrum compression axis at the onset of the caudal fin (Fig. 4, blue arrows; Fig. 6) makes the vertebral column suddenly bendable at the peduncle joint, thus adjustment of the angle of attack of the caudal fin is possible. As previously argued^9,10^, the change of compression axis also stabilizes the tail stem while facilitating the caudal fin to be flattened. The rapid shortening of the centrum near the peduncle joint leads to an increased number of intervertebral joints in this region, whereas the decrease in the centrum shortening rate in the anterior caudal fin reduces the number of vertebral joints in the area. This skeletal arrangement is interpreted to make the peduncular region more flexible and more posterior region less flexible than otherwise. The tailbend formed by a series of conspicuously wedge-shaped centra physically fixes the angle of the caudal fin relative to the body axis. Without these wedges, the angle can change in heterocercal and hypocercal tails through bending of the vertebral column near the onset of the caudal fin by the rotational component of the propulsive force from the fin.

**Table 1:** Presence (+) or absence (−) of vertebral features associated with thunniform body plans in tunas (T), cetaceans (C), lamnid sharks (LS), and thunnosaurian ichthyosaurs (TI).

Morphology	Inferred function	T	C	LS	TI
Thick vertebral column for body length (Fig. 4J)	Stabilization of anterior body axis	+	+	+	+
Centrum widening before peduncle joint and abrupt narrowing after (Fig. 2, green arrows)	Peduncle stability and peduncle joint flexibility	(+)	+	+	+
Regional concentration of intervertebral joints around peduncle joint (Fig. 2, blue arrows)	Flexibility of peduncle joint	+	+	+	+
Posterior dislocation of rapid centrum size decrease in tail stem (Fig. 4, blue dotted lines)	Posterior concentration of tail oscillation	−	+	+	+
Slight lengthening of centrum after peduncle joint (Fig. 2C, D, peaks next to blue arrows)	Stabilization of anterior caudal fin through joint number reduction	−	(+)	+	+
Tailbend with a series of conspicuously wedge-shaped centra (Fig. 3)	Stabilization of tailbend angle and thus the caudal fin orientation	−	−	+	+

The skeletal features pointed out in this study (Table 1) would allow identifications of thunniform swimmers in the fossil record and studies of the evolution of thunniform swimming in lineages with fossil evidence. For example, some early lamniform sharks from the Cretaceous are known to have calcified vertebral columns preserved^16–19^, even including a caudal fin skeleton with hypochordal rays calcified as in lamnids^20^, enabling the examination of the possible thunniform features. Based on published data, at least *Squalicorax falcatus* seems to have a mixture of thunniform and non-thunniform features. The trunk vertebral column is thicker than 2.5% of the neck to caudal fluke length as in thunniform swimmers although the tail-stem vertebral column is not as thick, according to published measurements^19^. A graph depicting changes of vertebral diameter along the vertebral column^18^, similar to blue and red curves in Fig. 3 here, suggests that the vertebral diameters decrease only slowly near the anterior tail stem region^19^, possibly suggesting a thunniform feature, yet the more critical shifts of centrum morphology in the peduncular region is not obvious. The vertebral diameter continuously decreases gradually in the tail stem, unlike in thunniform swimmers.

Another cohort of fossil vertebrates that can be studied are Mesozoic marine reptiles, at least three of which gave rise to hypocercal caudal fins and were fully aquatic^21^, namely ichthyosaurs, mosasaurs^22^, and thalattosuchians^23^. Many non-thunniform species are known among ichthyosaurs basal to Thunnosauria^24,25^ with their skeletons preserved in articulation. Quantification of vertebral dimensions in these specimens would allow the tempo and mode of thunniform evolution in this lineage to be analyzed. For the other two lineages, possibilities of thunniform swimming have been minimally discussed so far. A preliminary investigation based on published data^14,22,26^ suggests that at least some mosasaurs were not thunniform swimmers. They lacked a series of conspicuously wedge-shaped centra despite the presence of some weakly wedge-shaped centra that do not form a consecutive series^14^ and instead had uniformly curved “tailbend,” somewhat resembling that in *Heterodontus* and *Carcharhinus*. Their caudal centra shortens constantly through the peduncular region unlike in thunniform swimmers, without slowing down in the anterior caudal fin (Fig. 4I). Their body proportions are more similar to those of Carcharhiniformes than to Lamnidae^22^. Metriorhynchid thalattosuchians possess conspicuously wedge-shaped tailbend centra. However, other features are poorly documented for the clade at this point, requiring future investigations.

The chronostratigraphic distribution of thunniform swimmers is heavily biased toward the more recent time period (Fig. 1). The first clade known to have given rise to this mode of swimming was ichthyosaurs—the oldest Thunnosauria dates back to the earliest Jurassic (about 201 million year ago—‘Mya’ hereafter), whereas Parvipelvia, a clade that contains Thunnosauria and similar forms, are known as early as the Norian of the Late Triassic (about 220 Mya)^24,27^. Whether or not non-thunnosaurian parvipelvians were thunniform swimmers needs to be investigated in the future. These earliest thunniform swimmers became extinct toward the end of the Cenomanian of the Late Cretaceous, coinciding with a series of global warming events^28^, before any of the three crown clades of thunniform swimmers were established. Whereas the clade Lamnidae is said to have evolved in the early Paleocene (up to about 66 Mya), the origin of the genus *Lamna* goes back at least to the early Pliocene^29^ (about 5 Mya) although it may be as early as in the late Miocene^30^ (up to about 11 Mya) or even in the Oligocene^29^ (upwards of roughly 30 Mya). The oldest record of a putative stem *Thunnus* is from the Eocene^31^ (about 39 Mya).

The absence of thunniform swimmers before the end-Permian mass extinction may appear puzzling given that Elasmobranchii, Actinopterygii, and Reptilia already existed before the extinction^32^. It may be hypothesized that the reason is ecological. All four clades of thunniform swimmers mainly feed on fast pelagic species of cephalopods and teleost fishes^33,34^, whereas these two prey clades diversified most drastically in the Jurassic and later time period. Most fossil actinopterygians from the Triassic and earlier time periods tend to be heavily built and covered with thick ganoid scales^35^, so the cruising ability of thunniform swimmers is probably not necessary to feed on these fishes. Similarly, although belemnites, an extinct clade of squid-like cephalopods, date back to the Late Triassic, fast swimming cephalopods were not very common until the Jurassic when belemnites diversified^36^. It is also noteworthy that the radiation of sharks, including lamniforms, during the onset of the Late Cretaceous^37,38^ coincides with when ichthyosaur diversity and disparity abruptly diminished, resulting in their eventual extinction by the end of the Cenomanian^28^. Whether there was a direct competition between the ichthyosaurs and lamniforms during the Cenomanian is uncertain. However, the extinction of ichthyosaurs likely opened up their thunniform niche, which could have concomitantly been filled by lamniform sharks that possessed at least a subset of thunniform features as discussed above. Therefore, the evolution of thunniform swimming may be tightly linked to the prey type evolution and, at least in part, niche availability through geologic time. However, further studies are warranted to test these ecological hypotheses in the future because it is yet unclear if the subsequent coexistence of tunas, lamnids, and neocetes in the Cenozoic indicates thunniform niche expansions through diversification of pelagic prey.

## Methods

A total of 63 extant shark species was examined, spanning nine orders and 23 families (Table S1). CT images of the sharks were downloaded through the links in a reference^13^. Vertebral centra were segmented from CT images in 3D Slicer^39^ and exported as 3D mesh files. For the 13 species listed in Table S1 as having individual centra calcified as separate disks or cylinders, each centrum was isolated and manually reoriented according to the anatomical orientations in Meshlab^40^. The length, width, and height of each calcified centrum were measured using meshlabserver 2019.08, with help from MultiMesh-Scripting 1.1 by Andrew Hazelden, and the resulting output log was converted to a table using R^41^.

Comparisons were made with seven cetaceans, three ichthyosaurs, and two scombrid fishes (Table S2). Cetacean measurements are based on published data (Table S2), *Scomber japonicus* was studied based on a CT scan available on MorphoSource.org (ark:/87602/m4/M85314), whereas the other specimens were measured directly using dial calipers.

The rates of centrum size change along the body were quantified in the following steps. A local regression—window of 15% (40% for ichthyosaurs) without smoothing—was fitted to each curve of centrum dimensions versus vertebral position, using locfit function in R^41^. Then, the first derivative of the regression, representing the rate of size change per centrum, was calculated for each vertebral position. The resulting rate curve was scaled to fit an arbitrary body proportion of 2:2:1 for the trunk, tail stem, and caudal fin in Fig. 2 to facilitate easier comparisons.

Differences of vertebral thickness was tested between two groups, thunniform swimmers (Thunnini, Lamnidae, Neoceti, and Thunnosauria) versus non-thunniform swimmers (the rest of the species in Table S2). Ratios were log-transformed to satisfy the assumption of normality in ANOVA.

### Supplementary Information

Supplementary Tables.

## Data Availability

All data are included in the Supplementary Information, except those found in cited publications.
