# Supplementary material for: Skeletal convergence in thunniform sharks, ichthyosaurs, whales, and tunas, and its possible ecological links through the marine ecosystem evolution
Source: Sci Rep. 2023 Oct 4;13:16664. doi: 10.1038/s41598-023-41812-z (PMC10550938; doi:10.1038/s41598-023-41812-z)
Supplement: Supplementary file 1 — Supplementary Tables. [file 41598_2023_41812_MOESM1_ESM.pdf]

Supplementary Information

Skeletal convergence in thunniform sharks, ichthyosaurs, whales, and tunas.

Ryosuke Motani[1,]* and Kenshu Shimada[2–4]

[1]Department of Earth and Planetary Sciences, University of California, Davis, One Shields Avenue, Davis, California 95616 USA. rmotani@ucdavis.edu

[2]Department of Environmental Science and Studies, DePaul University, 1110 West Belden Avenue, Chicago, Illinois 60614, USA, kshimada@depaul.edu

[3]Department of Biological Sciences, DePaul University, 2325 North Clifton Avenue, Chicago, Illinois 60614, USA

[4]Sternberg Museum of Natural History, Fort Hays State University, Hays, Kansas 67601, USA

*Corresponding author.

Table of Contents

Supplementary Table S1. Shark species examined in the present study. 2

Supplementary Table S2. List of species used in comparison, with respective measurements of the maximum centrum widths and lengths of the vertebral column. 5

Supplementary Table S3. Vertebral measurements from CT scan images used in Figs. 2 and 4. · 7

Table S1. Shark species examined in the present study. Specimen numbers are based on Kamminga et al. (2017)[13].

Order	Family	Genus	Species	Common name	Specimen
Carcharhiniformes	Carcharhinidae	*Carcharhinus*	*amboinensis*	Pigeye shark	RMNH.PISC.4264
		C.	*dussumieri*	Whitecheek shark	RMNH.PISC.7375
		C.	*falciformes*	Silky shark	ZMA.PISC.108686
		C.	*hemiodon*	Pondicherry shark	ZMA.PISC.109135
		C.	*leucas*	Bull shark	RMNH.PISC.24271
		C.	*malcoti*	Hardnose shark	RMNH.PISC.7373
		C.	*melanopterus*	Blacktip reef shark	ZMA.PISC.108682
		Isogomphodon	*oxyrhinchus*	Daggernose shark	ZMA.PISC.111240
		Prionace	*glauca*	Blue shark	ZMA.PISC.116203
		Rhizoprionodon	*terraenovae*	Atlantic sharpnose shark	ZMA.PISC.111362
		Scoliodon	*laticaudus*	Spadenose shark	RMNH.PISC.8574
		Triaenodon	*obesus*	Whitetip reef shark	ZMA.PISC.110760
	Hemigaleidae	*Chaenogaleus*	*macrostomata*	Hooktooth shark	RMNH.PISC.7389.a
		Hemigaleus	*microstomata*	Sicklefin weasel shark	ZMA.PISC.109157
	Scyliorhinidae	*Apristurus*	*laurussonii*	Iceland catshark	BMNH1987.1.21.3
		Atelomycterus	*marmoratus*	Coral catshark	RMNH.PISC.34027
		Galeus	*melastomus*	Blackmouth catshark	RMNH.PISC.23617
		Halaelurus	*bosemani*	Speckled catshark	RMNH.PISC.34025
		Scyliorhinus	*boa*	Boa catshark	ZMA.PISC.140166
		S.	*canicula*	Small-spotted catshark	ZMA.PISC.111399.a
		S.	*stellaris*	Nursehound	RMNH.PISC.34047.f
	Sphyrnidae	*Eusphyra*	*blochii*	Winghead shark	RMNH.PISC.4204
		Sphyrna	*corona*	Scalloped bonnethead	RMNH.PISC.37210
		S.	*leweni*	Scalloped hammerhead	RMNH.PISC.7393.d
		S.	*tiburo*	Bonnethead	RMNH.PISC.4212
		S.	*tudes*	Smalleye hammerhead	ZMA.PISC.109128
		S.	*zygaena*	Smooth hammerhead	ZMA.PISC.108725
	Triakidae	*Galeorhinus*	*galeus*	School shark	RMNH.PISC.23690

		Mustelus	*asterias*	Starry smooth-hound	ZMA.PISC.120789
		M.	*higmani*	Smalleye smooth-hound	RMNH.PISC.23320
		M.	*mustelus*	Common smooth-hound	ZMA.PISC.109497
		Triakis	*semifasciata*	Leopard shark	ZMA.PISC.114250
Heterodontiformes	Heterodontidae	*Heterodontus*	*francisci*	Horn shark	ZMA.PISC.108688
		H.	*japonicus*	Japanese bullhead shark	RMNH.PISC.4202
Hexanchiformes	Hexanchidae	*Heptranchias*	*perlo*	Sharpnose sevengill shark	ZMA.PISC.111361
		Chlamydoselachus	*anguineus*	Frilled shark	BMNH1935.2.26.1
Lamniformes	Alopiidae	*Alopias*	*vulpinus*	Common thresher	ZMA.PISC.111198
	Lamnidae	*Lamna*	*nasus*	Porbeagle	ZMA.PISC.116165
Orectolobiformes	Brachaeluridae	*Brachaelurus*	*waddi*	Blind shark	BMNH1890.9.23.233
	Ginglymostomatidae	*Ginglymostoma*	*cirratum*	Nurse shark	ZMA.PISC.108710
		Nebrius	*ferrugineus*	Tawny nurse shark	ZMA.PISC.114675
	Hemiscyllidae	*Chiloscyllium*	*arabicum*	Arabian carpetshark	ZMA.PISC.140443
		C.	*indicum*	Slender bamboo shark	ZMA.PISC.114781
		Hemiscyllium	*strahani*	Hooded carpetshark	RMNH.PISC.33994
		H.	trispeculare	Speckled carpetshark	RMNH.PISC.35295.ab
	Orectolobidae	*Eucrossorhinus*	*dasypogon*	Tasselled wobbegong	RMNH.PISC.7411
		Orectolobus	*japonicus*	Japanese wobbegong	BMNH1982.11.1.90
	Stegostomatidae	*Stegostoma*	*fasciatum*	Zebra shark	RMNH.PISC.7401.b
Pristiophoriformes	Pristiophoridae	*Pristiophorus*	*japonicus*	Japanese sawshark	BMNH1936.7.29.12
Squaliformes	Centrophoridae	Centrophorus	seychellorum	Seychelles gulper shark	BMNH1973.7.9.13
		Deania	*calcea*	Birdbeak dogfish	RMNH.PISC.37981
	Dalatiidae	*Dalatias*	*licha*	Kitefin shark	ZMA.PISC.112272
		Euprotomicrus	*bispinatus*	Pygmy shark	RMNH.PISC.17804
		Isistius	*brasiliensis*	Cookiecutter shark	RMNH.PISC.37689
	Etmopteridae	*Etmopterus*	*spinax*	Velvet belly lanternshark	RMNH.PISC.21558.b
	Oxynotidae	*Oxynotus*	*centrina*	Angular roughshark	ZMA.PISC.110745
	Somniosidae	*Centroscymnus*	*crepidater*	Longnose velvet dogfish	BMNH2000.2.24.4
	Squalidae	*Squalus*	*acanthias*	Spiny dogfish	ZMA.PISC.116130
		S.	*cubensis*	Cuban dogfish	ZMA.PISC.111207

Squatiniformes	Squatinidae	*Squatina*	*africana*	African angelshark	ZMA.PISC.108696
		S.	*japonica*	Japanese angelshark	ZMA.PISC.113049
		S.	*squantina*	Angelshark	ZMA.PISC.115140
Echinorhiniformes	Echinorhinidae	*Echinorhinius*	*brucus*	Bramble shark	BMNH1900.11.6.7

Table S2. List of species used in comparison, with respective measurements of the maximum centrum widths and lengths of the vertebral column.

Clade	Species	Thunniform	Total tail-stock centrum lengths (mm)	Maximum tail-stock centrum width (mm)*	Ratio	Total centrum length from neck to caudal fin onset	Maximum centrum width from neck to caudal fin onset	Ratio	Specimen # or reference
Ichthyosauria	*Chaohusaurus brevifemoralis*	N	232	11.8	5.1%	548	12	2.2%	AGB6258, Anhui Geological M
Ichthyosauria	*Ophthalmosaurus icenicus*	Y	709	81.0	11.4%	1931	81	4.2%	PMAG R340** Peterborough
Ichthyosauria	*Ophthalmosaurus icenicus****	Y	1229	110.8	9.0%	2945	111	3.8%	NHMUK R3893, Natural Histo
Ichthyosauria	*Stenopterygius quadriscissus*	Y	837	62.2	7.4%	1945	62	3.2%	SMNS 14846, Staatliches Mus
Neoceti	*Balaenoptera acutorostrata*	Y	1756	157	8.9%	4855	157	3.2%	Ref. [41]
Neoceti	*Balaenoptera acutorostrata****	Y	1230	117.0	9.5%	3430	117	3.4%	Ref. [41]
Neoceti	*Balaenoptera edeni*	Y	2905	238	8.2%	8743	238	2.7%	Ref. [42]
Neoceti	*Eubalaena japonica*	Y	2561	325	12.7%	6469	325	5.0%	Ref. [43]
Neoceti	*Feresa attenuata*	Y	551	49	8.9%	1327	49	3.7%	Ref. [44]
Neoceti	*Mesoplodon ginkgodens*	Y	1332	93	7.0%	3187	93	2.9%	Ref. [45]
Neoceti	*Mesoplodon stejnegeri*	Y	1214	103	8.5%	3217	103	3.2%	Ref. [46]
Neoceti	*Physeter macrocephalus*	Y	2485	336	13.5%	5300	336	6.3%	Ref. [40]
Neoselachii	*Alopias vulpinus*	N	175.9	12.9	7.3%	491.2	12.9	2.6%	Ref. [13]
Neoselachii	*Carcharhinus leucas*	N	205.0	11.5	5.6%	558.2	11.5	2.1%	Ref. [13]
Neoselachii	*Galeorhinus galeus*	N	443.5	26.8	6.0%	957.2	21.1	2.2%	Ref. [13]
Neoselachii	*Heterodontus francisci*	N	271.5	14.9	5.5%	380.4	7.6	2.0%	Ref. [13]
Neoselachii	*Lamna nasus*	Y	198.6	7.6	3.8%	1078.9	26.8	2.5%	Ref. [13]
Neoselachii	*Sphyrna tudes*	N	531.6	20.9	3.9%	648.1	14.9	2.3%	Ref. [13]
Neoselachii	*Triakis semifasciata*	N	384.0	21.1	5.5%	1034.3	20.9	2.0%	Ref. [13]
Scombridae	*Euthynnus alletteratus*	Y	285.7	18.7	6.5%	504.3	18.7	3.7%	ROM R1487, Royal Ontario M
Scombridae	*Scomber japonicus*	N	43.1	1.6	3.7%	73.9	1.6	2.2%	SIO 80-267, Scripps Institute o

*maximum centrum height in neocetes
**centrum lengths are underestimated due to severe taphonomic compaction
***excluded from ANOVA and boxplot

Supplementary References

41 Omura H. 1957 Osteological study of the little piked whale from the coast of Japan. *Sci. Reports Whales Res. Inst.* 12, 1–21, 8 pls.

42 Omura H. 1959 Bryde's whale from the coast of Japan. *Sci. Reports Whales Res. Inst.* 14, 1–34.

43 Omura H. 1958 North Pacific right whale. *Sci. Reports Whales Res. Inst.* 13, 1–52.

44 Yamada M. 1954 An account of a rare porpoise, *Feresa* Gray from Japan. *Sci. Reports Whales Res. Inst.* 9, 59–88.

45 Nishiwaki M, Kamiya T. 1958 A beaked whale *Mesoplodon* stranded at Oiso beach, Japan. *Sci. Reports Whales Res. Inst.* 13, 53–84.

46 Nishiwaki M, Kamiya T. 1959 *Mesoplodon stejnegeri* from the coast of Japan. *Sci. Reports Whales Res. Inst.* 14, 35–48.

Table S3. Vertebral measurements from CT scan images used in Figs. 2 and 4.

Abbreviations: Vn, vertebral number; L, centrum length; W, centrum width; H, vertebral height.

Carcharhinus leucas

Vn	L	W	H	RMNH.PISC.24271
1	5.10	7.93	7.78	
2	4.77	8.37	8.45	
3	4.31	8.85	8.90	
4	4.20	10.03	9.28	
5	4.40	10.00	9.29	
6	4.48	10.00	9.49	
7	4.60	9.76	9.66	
8	4.55	9.67	9.76	
9	4.69	9.73	9.56	
10	4.77	9.79	9.69	
11	4.64	9.83	9.67	
12	4.64	9.90	9.84	
13	4.90	9.89	9.80	
14	4.92	9.97	9.88	
15	5.29	9.96	10.13	
16	5.08	10.04	10.12	
17	5.17	10.03	10.25	
18	5.20	10.30	10.25	
19	5.07	10.24	10.22	
20	5.11	10.34	10.31	
21	5.00	10.36	10.54	
22	5.01	10.51	10.40	
23	5.16	10.44	10.43	
24	5.09	10.57	10.51	
25	4.94	10.66	10.45	
26	4.97	10.77	10.47	
27	5.23	10.81	10.56	
28	5.06	10.82	10.56	
29	5.01	10.71	10.73	
30	5.02	10.81	10.70	
31	5.12	10.99	10.69	
32	5.12	10.88	10.77	
33	5.19	10.83	10.66	
34	5.18	10.86	10.96	
35	5.25	11.00	10.94	
36	5.14	10.81	10.78	
37	5.24	10.92	10.84	
38	5.19	10.90	10.95	

39	5.09	10.96	10.99
40	5.24	10.85	11.01
41	5.23	10.82	10.95
42	5.24	10.95	10.99
43	5.38	11.11	11.07
44	5.44	11.07	11.04
45	5.26	10.91	11.14
46	5.39	10.99	11.12
47	5.37	10.98	11.16
48	5.38	11.19	11.25
49	5.51	11.23	11.17
50	5.57	11.16	10.95
51	5.46	11.14	11.26
52	5.59	11.24	11.23
53	5.54	11.08	11.20
54	5.74	11.29	11.33
55	5.95	11.26	11.37
56	5.94	11.39	11.39
57	5.97	11.33	11.33
58	6.09	11.34	11.35
59	6.14	11.46	11.65
60	6.03	11.45	11.35
61	6.20	11.28	11.31
62	6.27	11.48	11.41
63	6.34	11.30	11.33
64	6.19	11.33	11.34
65	6.06	11.21	11.27
66	5.95	11.30	11.30
67	5.83	10.91	11.08
68	5.42	10.85	11.03
69	5.21	10.58	11.02
70	4.81	10.53	10.84
71	4.62	10.49	10.55
72	4.73	10.42	10.55
73	4.69	10.32	10.72
74	4.44	10.25	10.55
75	4.62	10.42	10.57
76	4.20	10.12	10.64
77	4.15	10.11	10.27
78	4.01	10.13	9.98
79	4.09	10.12	10.04
80	4.05	9.97	9.81
81	4.21	9.92	9.92

82	4.16	9.70	9.95
83	4.09	9.81	9.88
84	4.05	9.85	9.83
85	3.97	9.86	10.00
86	4.04	9.72	10.02
87	4.03	9.87	9.92
88	3.95	9.82	9.81
89	4.15	9.61	9.83
90	4.19	9.67	9.69
91	4.19	9.64	9.65
92	4.00	9.62	9.65
93	4.01	9.59	9.87
94	4.14	9.66	9.64
95	3.97	9.58	9.42
96	4.02	9.63	9.71
97	4.03	9.50	9.72
98	3.97	9.40	9.72
99	4.10	9.28	9.43
100	3.90	9.48	9.29
101	4.03	9.35	9.41
102	3.88	9.26	9.22
103	4.20	9.16	9.16
104	3.86	9.22	9.17
105	3.92	8.99	9.15
106	3.88	9.13	9.01
107	3.78	9.22	8.85
108	3.82	9.05	9.14
109	4.01	9.01	9.11
110	4.00	9.13	9.09
111	3.74	9.09	9.03
112	3.68	9.03	8.94
113	3.61	9.09	8.75
114	3.56	8.98	8.57
115	3.75	8.93	8.71
116	3.59	8.94	8.82
117	3.56	8.92	8.73
118	3.53	8.74	8.69
119	3.46	8.80	8.42
120	3.31	8.61	8.44
121	3.50	8.35	8.69
122	3.22	8.30	8.71
123	3.17	8.37	8.42
124	3.16	8.38	8.26

125	3.22	8.52	8.38
126	3.24	8.53	8.17
127	3.20	8.34	8.11
128	3.13	8.30	7.91
129	3.06	8.09	8.05
130	2.98	8.08	7.96
131	3.04	8.22	7.71
132	3.09	8.15	7.95
133	3.00	8.15	7.78
134	2.99	7.88	7.57
135	3.01	7.81	7.64
136	3.01	8.09	7.41
137	2.81	8.03	7.48
138	2.84	7.77	7.43
139	2.90	7.57	7.48
140	3.00	7.42	7.32
141	2.70	7.41	7.05
142	2.70	7.61	7.14
143	2.69	7.28	7.06
144	2.65	7.30	7.32
145	2.65	7.23	6.90
146	2.61	7.01	6.95
147	2.70	6.93	6.94
148	2.53	7.05	6.63
149	2.63	6.90	6.71
150	2.49	6.73	6.68
151	2.60	6.78	6.29
152	2.49	6.82	6.41
153	2.38	6.62	6.38
154	2.35	6.19	6.10
155	2.35	6.10	6.15
156	2.25	6.23	5.81
157	2.52	6.08	5.97
158	2.25	6.11	5.84
159	2.29	6.02	5.78
160	2.08	5.74	5.80
161	2.18	5.68	5.48
162	2.01	5.87	5.66
163	2.27	5.74	5.50
164	2.24	5.31	5.29
165	1.88	5.33	5.10
166	1.94	5.39	4.98
167	2.13	5.34	5.02

168	2.04	5.16	4.98
169	1.98	4.92	4.85
170	1.99	4.69	4.82
171	1.73	4.89	4.55
172	2.01	5.01	4.64
173	2.06	4.81	4.55
174	2.03	4.52	4.52
175	2.22	4.55	4.51
176	1.75	4.27	4.16
177	1.85	4.36	4.04
178	1.77	3.99	3.67
179	1.69	3.79	3.48
180	1.86	3.69	3.56
181	1.64	3.57	3.55
182	1.75	3.62	3.69
183	1.50	3.53	3.32
184	1.75	3.52	3.45
185	1.51	3.33	3.29
186	1.54	3.20	2.68
187	1.31	3.13	3.18
188	1.53	3.06	3.03
189	1.12	3.18	2.67
190	0.84	2.58	2.15
191	0.91	2.80	2.14
192	1.55	2.46	2.70
193	1.52	2.34	2.58
194	1.48	2.22	2.46
195	1.44	2.10	2.35
196	1.40	1.99	2.23
197	1.36	1.87	2.11
198	1.33	1.75	1.99
199	1.29	1.63	1.88
200	1.25	1.51	1.76
201	1.21	1.39	1.64
202	1.18	1.28	1.53
203	1.14	1.16	1.41
204	1.10	1.04	1.29
205	1.06	0.92	1.18
206	1.00	1.30	1.50

Euthynnus alletteratus

Vn	L	W	H	ROM R1487
1	8.7	13.4	12.7	

2	8.8	12.3	12.1
3	10.2	13.6	11.4
4	9.8	14.4	10.7
5	9.5	14.4	10.1
6	10.4	13.3	10.1
7	10.9	12.6	11.2
8	11.8	12.7	11.1
9	11.6	12.9	11
10	11.5	13.3	11.3
11	11.8	13.1	11.6
12	11.5	13.2	11.6
13	12.5	13.4	11.6
14	12.1	13.6	11.8
15	13.2	13.9	11.6
16	12.9	14	12
17	13.9	14	12.2
18	13.6	14.1	12.2
19	13.9	14.4	12.6
20	14.3	14.5	12.7
21	14.5	14.5	12.9
22	15.2	15.5	12.9
23	15.5	15	13.2
24	15.8	15	13.1
25	16.6	15.6	13.5
26	16	15.4	13.5
27	17	15.5	13.6
28	18.1	15.8	14.1
29	19.1	16.1	13.9
30	19.6	16.8	14.5
31	20.9	17.3	14.9
32	22.8	18	14.7
33	23.4	18.7	14.6
34	21.6	17.9	12.5
35	15.3	14.5	10
36	10.1	11.1	8.5
37	5.5	9	7.8
38	4.5	7.9	8.4

Galeorhinus_galeus

Vn	L	W	H	RMNH.PISC.23690
1	10.24	21.15	12.19	
2	9.49	14.05	12.45	
3	9.77	13.81	12.69	

4	9.80	13.83	13.16
5	9.66	14.05	13.19
6	9.88	14.15	13.29
7	10.13	14.12	13.56
8	10.54	14.40	14.09
9	10.80	14.91	13.97
10	10.80	15.06	14.11
11	10.88	15.59	14.58
12	11.54	15.68	14.47
13	11.69	16.10	14.55
14	12.17	16.47	15.38
15	12.18	16.44	15.14
16	12.45	16.78	15.50
17	12.36	16.74	15.40
18	13.15	17.27	15.50
19	13.00	17.12	15.52
20	13.07	17.13	15.67
21	13.49	17.13	15.71
22	13.60	17.42	15.82
23	14.53	17.54	16.00
24	14.21	17.46	15.96
25	14.91	17.28	15.81
26	14.82	17.29	15.93
27	14.97	17.39	15.97
28	15.40	17.95	15.81
29	15.43	17.43	16.18
30	16.32	17.37	16.51
31	16.58	17.48	16.60
32	17.09	17.74	16.62
33	17.55	17.88	16.56
34	17.95	18.05	16.74
35	18.89	18.22	17.26
36	19.41	18.29	17.32
37	20.52	18.59	17.51
38	20.44	19.25	17.48
39	20.14	19.11	17.65
40	18.31	18.17	17.23
41	14.93	17.36	16.66
42	16.10	16.73	15.74
43	13.18	16.58	15.40
44	11.86	15.77	15.26
45	14.03	15.52	15.27
46	10.30	15.42	15.04

47	13.72	15.31	14.95
48	10.79	15.57	15.42
49	13.15	15.29	14.89
50	9.93	15.18	14.63
51	13.05	15.32	14.62
52	9.81	14.98	14.36
53	12.46	14.88	14.26
54	9.47	14.80	14.07
55	12.07	14.90	13.99
56	9.15	14.77	13.84
57	11.37	14.51	13.67
58	9.08	14.57	13.60
59	10.86	14.42	13.47
60	8.08	14.40	13.27
61	10.78	14.04	13.10
62	7.85	14.02	13.08
63	10.32	13.88	12.82
64	7.81	13.33	12.94
65	10.00	13.45	13.07
66	7.83	13.57	13.04
67	9.27	13.67	12.84
68	8.18	13.18	12.57
69	9.20	13.43	12.59
70	7.51	13.21	12.58
71	8.97	13.17	12.40
72	7.73	13.12	12.58
73	8.72	12.82	12.25
74	7.02	13.38	12.23
75	7.67	13.06	12.70
76	7.13	12.89	12.53
77	8.45	13.01	12.54
78	6.69	13.22	12.25
79	8.48	13.16	12.73
80	5.98	13.24	12.53
81	7.71	12.94	12.33
82	5.93	12.71	12.59
83	7.58	12.46	12.22
84	5.76	12.33	12.13
85	7.59	12.36	11.64
86	6.05	12.51	11.90
87	6.89	11.97	11.41
88	5.49	11.62	11.07
89	6.47	11.49	10.95

90	5.73	11.02	11.65
91	6.59	11.08	10.61
92	5.36	10.82	10.32
93	6.51	11.04	10.76
94	5.40	10.50	10.39
95	5.82	10.78	9.99
96	5.19	9.98	9.62
97	5.66	10.06	9.40
98	4.83	9.89	9.27
99	5.32	9.92	9.47
100	5.26	9.37	8.55
101	4.97	8.99	9.05
102	5.20	9.12	8.48
103	4.54	8.91	8.42
104	5.28	8.74	8.49
105	5.03	8.71	8.07
106	4.86	8.29	8.19
107	5.27	7.98	8.15
108	4.22	7.62	7.37
109	4.52	7.53	7.80
110	4.55	7.18	6.88
111	4.61	6.67	6.93
112	3.82	5.96	6.73
113	3.99	6.46	6.64
114	4.05	5.84	5.91
115	5.02	5.53	5.08
116	3.61	5.15	4.41
117	4.60	4.22	3.23
118	3.67	3.83	3.64
119	3.26	1.01	3.51
120	2.23	1.07	2.62

Lamna nasus

Vn	L	W	H	ZMA.PISC.116165
1	8.69	17.47	15.85	
2	8.49	17.90	17.08	
3	8.84	18.59	17.70	
4	9.26	19.37	18.16	
5	9.43	19.79	18.71	
6	9.86	19.86	18.97	
7	10.10	20.64	19.39	
8	10.26	21.02	19.94	
9	10.44	21.06	20.30	

10	10.48	21.20	20.69
11	10.44	21.20	20.69
12	10.44	21.09	21.12
13	10.25	21.48	21.17
14	10.14	21.64	21.16
15	10.10	21.47	21.45
16	10.45	21.50	21.68
17	10.59	21.52	21.81
18	10.41	21.59	21.57
19	10.59	21.67	21.57
20	10.39	21.96	21.49
21	10.22	21.90	21.78
22	9.91	22.19	21.70
23	10.55	22.41	22.19
24	10.48	22.99	22.39
25	10.47	23.05	21.94
26	10.28	23.05	23.08
27	10.13	23.17	23.25
28	10.24	22.90	23.99
29	10.50	22.81	23.71
30	10.49	22.96	24.21
31	10.65	23.43	24.21
32	11.03	23.34	24.91
33	11.28	23.77	25.19
34	11.27	24.10	25.09
35	11.62	24.41	25.16
36	11.94	24.54	25.25
37	12.02	24.80	25.04
38	11.69	24.36	24.88
39	12.00	24.50	25.31
40	12.20	24.62	25.40
41	12.10	25.26	25.31
42	12.90	25.53	25.39
43	12.87	25.43	25.42
44	13.02	25.68	25.20
45	13.80	25.96	25.42
46	13.91	25.69	25.73
47	14.35	25.78	25.88
48	14.57	25.73	26.35
49	14.86	25.72	26.24
50	15.25	25.67	26.30
51	15.40	26.17	26.70
52	15.54	26.01	26.89

53	15.74	26.18	27.28
54	15.91	26.29	27.07
55	16.52	26.60	27.56
56	15.89	26.40	27.30
57	15.88	26.45	27.44
58	14.68	26.13	27.28
59	13.50	26.23	27.30
60	13.15	26.45	27.46
61	13.15	26.46	27.25
62	13.40	26.49	27.16
63	13.21	26.65	27.08
64	13.35	26.53	27.15
65	13.80	26.55	27.19
66	14.46	26.68	27.01
67	14.12	26.80	27.07
68	13.84	26.26	26.73
69	13.76	26.12	26.73
70	14.11	26.42	26.34
71	14.36	25.85	26.46
72	12.56	26.07	25.86
73	11.64	25.74	25.43
74	12.74	25.65	25.63
75	12.86	25.73	25.46
76	13.85	25.66	25.07
77	13.75	25.11	24.79
78	13.60	24.64	24.02
79	12.71	23.86	23.52
80	12.20	23.43	22.93
81	11.39	22.98	22.40
82	11.45	22.67	21.92
83	10.92	21.90	21.53
84	11.18	21.84	21.07
85	10.95	21.56	21.10
86	10.08	21.52	20.72
87	10.19	21.16	20.65
88	9.55	21.02	20.30
89	8.68	20.53	19.56
90	8.53	20.35	19.43
91	8.27	19.92	18.89
92	8.11	19.38	18.52
93	7.73	18.79	18.34
94	7.98	18.11	17.69
95	8.16	17.37	17.34

96	8.20	16.75	17.05
97	7.79	16.53	16.66
98	7.97	16.49	17.07
99	8.25	16.43	17.32
100	7.90	16.52	16.86
101	8.11	16.37	17.09
102	7.63	16.49	16.65
103	7.92	16.56	16.57
104	8.50	16.42	16.02
105	8.19	16.64	16.06
106	8.44	16.26	15.54
107	8.27	16.03	15.35
108	8.75	15.90	15.07
109	8.15	15.70	15.04
110	8.46	15.31	14.87
111	8.33	14.66	14.70
112	7.72	14.60	14.22
113	7.89	14.42	14.17
114	7.82	13.92	13.89
115	8.36	13.69	13.93
116	7.72	13.37	13.57
117	7.56	13.05	13.28
118	7.40	12.74	12.98
119	7.25	12.42	12.69
120	7.09	12.10	12.39
121	6.93	11.79	12.10
122	6.77	11.47	11.80
123	6.61	11.16	11.51
124	6.45	10.84	11.22
125	6.29	10.52	10.92
126	6.13	10.21	10.63
127	5.97	9.89	10.33
128	5.81	9.57	10.04
129	5.66	9.26	9.74
130	5.50	8.94	9.45
131	5.34	8.63	9.16
132	5.18	8.31	8.86
133	5.02	7.99	8.57
134	4.86	7.68	8.27
135	4.70	7.36	7.98
136	4.54	7.05	7.69
137	4.38	6.73	7.39
138	4.23	6.41	7.10

139	4.07	6.10	6.80
140	3.70	6.60	7.10
141	3.59	6.21	6.79
142	3.48	5.82	6.48
143	3.37	5.43	6.17
144	3.26	5.04	5.86
145	3.15	4.65	5.55
146	3.04	4.26	5.24
147	2.93	3.87	4.93
148	2.82	3.48	4.62
149	2.71	3.09	4.31
150	2.60	2.70	4.00
151	2.44	2.54	3.78
152	2.28	2.38	3.56
153	2.12	2.22	3.34
154	1.96	2.06	3.12
155	1.80	1.90	2.90
156	1.64	1.74	2.68
157	1.48	1.58	2.46
158	1.32	1.42	2.24
159	1.16	1.26	2.02
160	1.00	1.10	1.80

Scomber japonicus

Vn	L	W	H
1	1.96	1.55	1.46
2	2.18	1.49	1.39
3	2.17	1.44	1.32
4	2.23	1.39	1.38
5	2.26	1.38	1.34
6	2.37	1.38	1.42
7	2.38	1.40	1.40
8	2.43	1.39	1.38
9	2.51	1.40	1.44
10	2.58	1.40	1.48
11	2.61	1.41	1.47
12	2.55	1.45	1.52
13	2.54	1.50	1.57
14	2.63	1.53	1.56
15	2.66	1.55	1.59
16	2.62	1.57	1.63
17	2.68	1.58	1.63
18	2.64	1.57	1.63

19	2.60	1.58	1.64
20	2.65	1.60	1.63
21	2.68	1.57	1.65
22	2.73	1.57	1.68
23	2.75	1.55	1.66
24	2.75	1.58	1.60
25	2.71	1.58	1.61
26	2.68	1.56	1.62
27	2.45	1.53	1.53
28	2.36	1.51	1.47
29	2.18	1.44	1.49
30	1.37	1.09	1.29

Sphyrna turdes

Vn	L	W	H	ZMA.PISC.109128
1	5.40	9.27	8.48	
2	4.52	9.19	9.04	
3	4.54	9.34	9.43	
4	4.85	9.64	9.30	
5	5.08	9.56	9.87	
6	5.30	9.68	9.87	
7	5.24	9.78	9.95	
8	5.57	9.88	10.25	
9	6.14	10.17	10.36	
10	5.91	9.96	10.48	
11	5.80	9.97	10.43	
12	5.74	10.23	10.38	
13	5.91	10.09	10.67	
14	5.87	10.27	10.68	
15	6.11	10.30	10.92	
16	6.17	10.55	10.82	
17	6.65	10.63	11.38	
18	6.38	10.55	11.44	
19	6.31	10.78	11.23	
20	6.51	10.76	11.39	
21	6.55	11.04	11.62	
22	6.69	11.25	11.53	
23	6.76	11.38	11.80	
24	6.83	11.43	11.86	
25	6.87	11.36	12.11	
26	7.02	11.65	12.12	
27	7.25	11.76	12.33	
28	7.13	11.61	12.31	

29	7.27	11.87	12.37
30	7.24	11.88	12.47
31	7.69	11.85	12.43
32	7.67	11.99	12.53
33	7.36	11.84	12.38
34	7.58	12.18	12.57
35	7.88	12.08	12.44
36	7.81	12.54	12.62
37	7.94	12.28	12.59
38	8.11	12.53	12.50
39	8.29	12.59	12.72
40	8.14	12.42	12.86
41	8.47	12.45	12.79
42	8.43	12.24	12.89
43	8.79	12.44	12.84
44	8.70	12.54	12.84
45	8.79	12.45	13.20
46	9.15	12.64	13.36
47	9.04	12.53	13.17
48	9.23	12.62	13.26
49	9.32	12.85	13.34
50	9.23	12.71	13.19
51	9.87	13.30	13.34
52	10.11	13.15	13.60
53	10.43	13.15	13.64
54	10.90	13.18	13.49
55	11.27	13.29	13.76
56	11.20	13.11	13.24
57	10.87	13.06	13.10
58	9.16	12.51	12.84
59	7.53	12.02	12.20
60	6.71	11.90	12.12
61	6.66	11.58	11.88
62	6.83	11.44	11.90
63	6.67	11.44	11.84
64	6.74	11.23	11.78
65	6.60	11.41	11.82
66	6.72	11.36	11.69
67	6.74	11.23	11.33
68	6.68	11.20	11.26
69	6.69	11.19	11.69
70	6.75	11.16	11.75
71	6.49	11.16	11.23

72	6.67	11.10	11.34
73	6.51	10.89	11.49
74	6.40	10.99	11.36
75	6.70	10.70	11.24
76	6.34	10.63	11.12
77	6.04	10.92	11.43
78	6.12	10.61	11.13
79	5.98	10.83	11.10
80	5.95	10.68	10.94
81	5.87	10.65	10.96
82	5.92	10.56	10.96
83	6.04	10.60	11.05
84	5.83	10.34	10.69
85	5.59	10.43	10.75
86	5.70	10.18	10.56
87	5.78	10.13	10.83
88	5.67	10.11	10.83
89	5.51	10.21	10.62
90	5.41	10.05	10.54
91	5.37	10.02	10.46
92	5.35	9.88	10.47
93	5.39	9.96	10.37
94	5.05	9.83	10.39
95	5.36	9.81	10.25
96	5.10	9.72	10.27
97	5.12	9.67	10.05
98	5.02	9.64	10.14
99	4.85	9.58	9.84
100	4.96	9.60	9.94
101	4.89	9.68	10.06
102	5.04	9.68	9.94
103	4.61	9.73	9.94
104	4.98	9.83	9.77
105	4.74	9.42	9.70
106	4.56	9.58	9.84
107	4.54	9.45	9.31
108	4.54	9.63	9.54
109	4.33	9.51	9.68
110	4.30	9.35	9.45
111	4.57	9.42	9.51
112	4.39	9.31	9.55
113	4.29	9.43	9.35
114	4.22	9.03	9.16

115	4.27	9.03	9.20
116	4.17	9.04	9.21
117	4.18	8.97	9.13
118	4.16	9.11	8.95
119	4.28	8.83	8.93
120	4.17	8.79	8.79
121	4.05	8.58	8.69
122	4.03	8.42	8.72
123	4.36	8.47	8.60
124	3.86	8.26	8.56
125	3.93	8.32	8.62
126	3.89	8.04	8.45
127	3.97	8.14	8.25
128	3.86	7.99	8.32
129	3.88	7.91	8.25
187	1.60	1.70	1.70
